# Highlights of Immunomodulation in *Salmonella*-Based Cancer Therapy

**DOI:** 10.3390/biomedicines9111566

**Published:** 2021-10-29

**Authors:** Christian R. Pangilinan, Che-Hsin Lee

**Affiliations:** 1 Department of Biological Sciences, National Sun Yat-sen University, Kaohsiung 80424, Taiwan; pangilchris@g-mail.nsysu.edu.tw; 2 Department of Medical Research, China Medical University Hospital, China Medical University, Taichung 40402, Taiwan; 3 Department of Medical Laboratory Science and Biotechnology, Kaohsiung Medical University, Kaohsiung 80708, Taiwan; 4 International PhD Program for Science, National Sun Yat-sen University, Kaohsiung 80424, Taiwan

**Keywords:** *Salmonella*, bacteria-mediated cancer therapy, immunomodulation, immunotherapy

## Abstract

Bacteria-mediated cancer therapy (BMCT) is an emerging tool that may advance potential approaches in cancer immunotherapy, whereby tumors are eradicated by the hosts’ immune system upon recruitment and activation by bacteria such as *Salmonella*. This paper provides an emphasis on the immunomodulatory effects that encompasses both the innate and adaptive immune responses inherently triggered by *Salmonella*. Furthermore, modifications of *Salmonella*-based treatment in the attempt to improve tumor-specific immune responses including cytokine therapy, gene therapy, and DNA vaccine delivery are likewise discussed. The majority of the findings described herein incorporate cell-based experiments and murine model studies, and only a few accounts describe clinical trials. *Salmonella*-based cancer therapy is still under development; nonetheless, the pre-clinical research and early-phase clinical trials that have been completed so far have shown promising and convincing results. Certainly, the continuous development of, and innovation on, *Salmonella*-based therapy could pave the way for its eventual emergence as one of the mainstream therapeutic interventions addressing various types of cancer.

## 1. Introduction

Cancer, though not necessarily hereditary, is a genetic disease characterized by an impaired control of cell cycle resulting in an unregulated cellular growth and proliferation. Despite the growth of decades-worth of findings on the nature, causes, and pathogenesis of cancer, it nevertheless retains its status as one of the leading causes of death worldwide. Today, the development of therapeutic strategies that involve directing a patient’s own immune system towards eradicating cancer offers a new hope to prolong survival of patients compared with conventional or standard care, i.e., chemotherapy, radiotherapy, and surgery [1]. The concept of using the immune system in cancer treatment can be traced back to the 19th century when Dr. William Coley developed a cocktail of heat-killed bacteria that became known as “Coley’s toxin” to cure a number of inoperable tumors [2]. The idea emerged when Dr. Coley found an old medical record where a patient suffering from an inoperable sarcoma was documented to have tumor regression following recovery from erysipelas infection (*Streptococcus pyogenes*); he soon found out that the patient was disease-free [2]. There were variable outcomes among all patients treated with Coley’s toxin at that time, nonetheless, some of the results were promising as some patients had complete clearance of the primary tumor while others were even declared disease-free [3]. Dr. Coley’s treatment strategy was not without issues, such as side effects, clearance of the bacterial infection, and the mechanisms on how the therapy specifically worked; consequently, this treatment strategy was not entirely recognized at that time and ultimately forgotten decades thereafter [2,3]. In recent years, however, advances in cancer immunobiology research paved the way to the further development of bacteria-mediated cancer therapy (BMCT) using different bacterial species such as *Streptococcus*, *Bacillus*, *Bifidobacterium*, *E. coli*, *Clostridium*, and *Salmonella*, among others [4].

In this review, we provide emphasis on the interplay between tumor and tumor-associated immune cells in the tumor microenvironment in the presence of BMCT intervention, particularly using *Salmonella* species. *Salmonella* are Gram negative, facultatively anaerobic, nonspore-forming rod-shaped enterobacteria studded with multiple flagella [5]. This species is preferred for this purpose because of its natural ability to address various types of cancer [6]. Immune modulation is of primary interest in this type of intervention since *Salmonella* is a microorganism that, upon successfully gaining access into a host, can trigger a cascade of immune responses. *Salmonella*-triggered immune responses with antitumor actions are of utmost importance in eliminating cancerous growth and preventing metastasis resulting in longer patient survival [1].

## 2. *Salmonella* as a Viable Option for Bacteria-Mediated Cancer Therapy

As scientists and clinicians continue to race in search for more effective and efficient treatments and therapeutic strategies against cancers, a growing interest on the use of *Salmonella* in this regard has emerged as one of the focal points in cancer research. One practical and interesting characteristic behavior of this bacteria is its capacity to selectively penetrate, favorably invade, and preferentially grow within tumors; a behavior that is made possible by chemotaxis, as they are attracted to the compounds and niche-specific conditions produced in the tumor microenvironment [7,8]. Furthermore, the facultatively anaerobic nature of *Salmonella* is advantageous, as they can tolerate the oxygen-depleted zone (i.e., about 10 to 30 mmHg concentration) of solid tumors. This ability is indispensable for successful dispersion of *Salmonella* to evenly exert its oncolytic and other anti-tumor progression effects via multiple mechanisms [9]. It is also noteworthy to mention that the dispersion of systemically administered *Salmonella* has a ratio of only 1000–10,000 to 1 tumor-to-normal tissue [10]. The ability of *Salmonella* to efficiently infect tumor cells is accounted for by its use of a type three secretion system (TTSS), since mutant *Salmonella* with defective TTSS exhibited substantial loss of tumor cell-penetrating potential [11]. Certain *Salmonella* strains have also been intentionally mutated to improve their tumor cell penetrating ability also via TTSS [12]. For these reasons, *Salmonella* species have been investigated for their potential use as vehicle for the direct delivery of drugs, RNA interferences, and cancer therapeutic genes into tumors and their innate potential to trigger various antitumor responses [13]. Apart from being an efficient tumor-targeting bacteria, when *Salmonella* infects tumor cells, it interferes with the tumor-intrinsic signaling cascade and modifies the overall pattern of gene expression, which, in turn, impacts cellular behavior leading to decreased migrative potential, decreased tumor survival, and stimulated antitumor immune responses [14]. The following sections will discuss further how *Salmonella*-based treatment modulates immune response against cancer.

## 3. Breaking the Immunosuppressive Nature of Tumor Microenvironment

**Figure 1:**
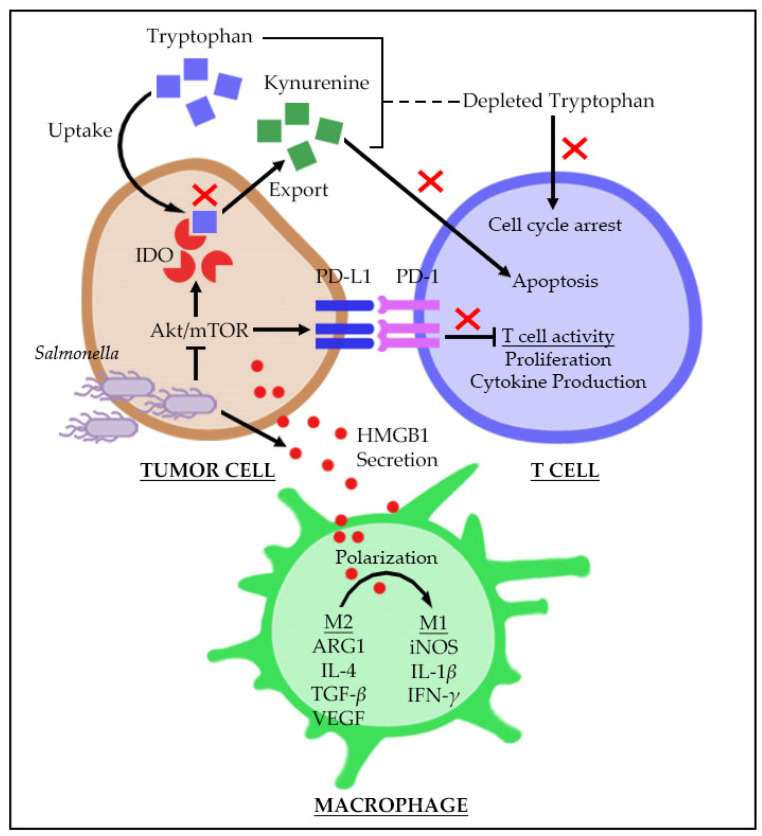
An overview of *Salmonella*-mediated modulation of tumor-specific immune responses in the tumor microenvironment. *Salmonella* overcomes the immunosuppressive TME by downregulating tumor-intrinsic IDO and PD-L1, leading to the reactivation and increase in the population of effector T cells. Further, *Salmonella* directs reprogramming of macrophages toward an antitumor phenotype upon stimulation by extracellular HMGB1. The symbol “X” shown in red means prevention/inhibition of the activity.

The tumor microenvironment (TME) harbors both cellular (immune infiltrates, fibroblasts, and endothelial cells) and non-cellular components (extracellular matrix or ECM, soluble molecules) that are present in the immediate surroundings of the tumor tissue [15,16], all of which interact to facilitate tumor growth and survival, and progression to metastasis. It has been suggested that the stromal composition in the TME may vary depending on the particular type and stage of tumor. Changes in the TME may affect the proliferative and metastatic potential of tumors, thus restricting or enabling progression [13]. Reports suggest that an immunosuppressive TME accounts for poor prognosis or clinical outcome among cancer patients [17,18,19]. Therefore, targeting the TME, particularly the priming of the immune cellular components inclined toward antitumor responses will be advantageous in eliminating cancer. Figure 1 illustrates a summary of the mechanisms involved in *Salmonella*-mediated modulation of immune responses in the TME.

Tumor molecular heterogeneity and tumor-intrinsic dysregulated signaling pathways contribute to the shaping of an inflammatory and immunosuppressive TME. For instance, Wnt/*β*-catenin activation in tumor cells from clinical samples mediates exclusion of tumor-specific T-cells from the TME [20]. Likewise, the activation of STAT3 signaling in tumor-implant models significantly decreased the expression of tumor-secretory proinflammatory mediators [21]. Another clinically relevant pathway that negatively impacts immune responses is the PI3K/PTEN/AKT pathway. PIK3CA activating mutations or PTEN loss-of-function mutations activate PI3K/AKT signaling that often results in an increase of immunosuppressive tumor-associated macrophage (TAM) infiltrates [22]. This signaling axis is also often involved in the regulation of tryptophan-degrading enzyme indoleamine-2,3-dioxygenase (IDO) in cancer and surrounding stroma [23,24]. High IDO activity deprives effector immune cells of available tryptophan and, in particular, T-cells undergo cell cycle arrest at the G1 phase due to depleted tryptophan [25,26]. Tryptophan deprivation can be sensed by T cells, thus triggering the kinase general control non-depressible 2 (GCN2) to undergo proliferative arrest, thereby increasing the Treg population [27,28]. Moreover, the byproduct of tryptophan catabolism, i.e., kynurenine, can induce T cell apoptosis [26]. Aberrant and high expression of immunosuppressive IDO has been determined to be a prognostic factor for poor clinical outcome [29,30] while the therapeutic inhibition of IDO may result in a favorable outcome. Interestingly, *Salmonella*-based treatment using *Salmonella enterica* serovar Choleraesuis significantly reduced IDO expression and activity by suppressing AKT signaling, specifically the AKT/mTOR/p70S6K axis [23]. Suppression of AKT/mTOR signaling by *Salmonella* was also found to downregulate the expression of tumor-intrinsic immune tolerance mediator, that is, programmed death-ligand1 (PD-L1) [31]—a transmembrane protein acting as a ligand for the immune checkpoint receptor PD-1, one of the gatekeepers of immune response, along with CTLA4, LAG3, TIM3, TIGIT and BTLA [32]. Effector T cell activation, TCR-mediated proliferation as well as cytokine production were expected to be suppressed with high expression of surface PD-L1 in tumors upon interaction with T cell PD-1 [33], which were reversed by *Salmonella* treatment [31]. In an earlier study, it was found that *Salmonella*-mediated tumor regression induced the phenotypic and functional activation of intratumoral myeloid cells, but not that of splenic CD11b+ cells, making them significantly less immunosuppressive in the TME; this observation was attributed to the significant reduction of IL-4-IL-13/ARG1 axis, a prototypical marker for immunosuppressive tumor-associated macrophages [34]. In another study, Flagellin from *Salmonella* was demonstrated to facilitate the breaking of immunosuppression in the TME by decreasing the population of CD4+CD25+ Treg cells in TME via TLR5 signaling [35].

## 4. Directing Immune Infiltrates toward Antitumor Responses

The immune cellular components of the TME are perceived to be critical in controlling tumorigenesis and progression to metastasis. While it is true that TME may harbor both tumor-promoting and tumor-suppressing innate and adaptive immune cells, the prevalence and ratio of these components predict clinical prognosis [20,36]. In most cases of cancer, the number of effector immune cells is overwhelmed by suppressive cells; however, these cells are not only suppressive, but they also provide the necessary element to support survival of tumors as well as progression to metastasis by mediating epithelial-to-mesenchymal transition (EMT), angiogenic sprouting, and extracellular matrix (ECM) remodeling [37,38]. As such, it is indeed feasible to set tumor-associated immune infiltrates as targets for cancer therapy. Strategizing on how to increase the antitumor immune activity was proven efficient against various cancer types including breast cancer [23], lung cancer [31,39], and melanoma [31], among others.

### 4.1. Adaptive Immunity

The antitumor capacity of *Salmonella* is not solely reliant on its own ability to induce tumor cell death as it can also promote a collaborative effort with effector cells recruited and activated within the TME. Our previous report suggested that systemic administration of *Salmonella enterica* serovar Choleraesuis exerts antitumor activity by stimulating T cell type 1 immune response as revealed by an increased interferon-*γ* (IFN-*γ*)-mediated response in wild type and CD8+ T cell-deficient mice as compared with CD4+ T cell-deficient mice [39]. Furthermore, we also found that *S. enterica* serovar Choleraesuis significantly up-regulated the production of IFN-*γ*, IFN-inducible chemokines CXCL9 (MIG), and CXCL10 (IP-10) in C3H/HeN wildtype, but not in C3H/HeJ TLR4-deficient mice, suggesting that TLR4 is involved in the stimulation of CD4+ and CD8+ T cell-dependent antitumor immune response [40]. Interestingly, another group showed that the accumulation of *Salmonella*-infected tumor cells undergoing tumor lysis may enable further stimulation of tumor-specific T cell responses, involving both CD4+ and CD8+ T cells, at a later time point post-treatment [11]. Cross-presentation of tumor-derived antigens from cellular debris is likely to cause the stimulation of naïve T cells [11]. Two other strains of *Salmonella,* namely LVR01 and 7207, were shown to induce recruitment of activated CD8+ T cells or cytotoxic T cells (CTL) accompanied by substantial increase in apoptotic and necrotic tumor cells [41,42]. Likewise, another strain of *Salmonella,* namely SalpNG.1, efficiently increased tumor-infiltrating CD8+ T cells and NK cells [43]. Another group suggested the involvement of *Salmonella* lipopolysaccharide (LPS) in the activation and recruitment of immune cells and the subsequent production of TNF-α resulting in tumor retardation [44]. Previously, we have explained a mechanism by which *Salmonella* increases activated tumor-specific T cells, which is by reducing tumor-intrinsic IDO expression (Figure 1) [23]. As mentioned, IDO depletes available tryptophan causing T cell arrest and increases kynurenine which is toxic to T cells [25]. The substantial downmodulation of IDO was caused, in part, by suppressing AKT/mTOR activity [23]. Furthermore, it has been shown that *Salmonella* may decrease IDO via another mechanism, i.e., by upregulating Cx43 [28], although the main role of Cx43 in inhibiting IDO expression remains unclear. Nevertheless, both suppression of the AKT/mTOR cascade and upregulation of Cx43 demonstrated a possible link among downmodulation of IDO, an increase in T cell infiltrates, and T cell-induced tumor apoptosis, particularly by CD8+ T cells. In a recent study from our group, we provided additional mechanisms to explain the increased reactivated tumor-specific T cell population (Figure 1). In particular, we showed that *Salmonella*-mediated downregulation of tumor PD-L1 contributes to the reactivation of tumor-specific T cells [31]. To evaluate the involvement of PD-L1 downregulation in increasing T cell activity, a coculture experiment was performed. Findings suggested that activated CD8+ T cell population was substantially increased while the number of T cells undergoing apoptosis was decreased; the outcome of the experiments were corroborated using tumor-bearing murine models [31]. A different *Salmonella* vaccine strain, namely *Salmonella enterica* serovar Typhimurium vaccine (RASV), also demonstrated a CD8+ T cell-dependent antitumor activity after intratumoral injection; however, the disparity in tumor volume between CD8+ T cell-depleted mice group with and without *Salmonella* treatment indicates other key players in the antitumor responses [45]. Although it was found that RASV treatment significantly increased CD11b(+)Gr-1(+) myeloid-derived suppressor cells (MDSC), a significant proportion of the these account for TNF-*α*-secreting Ly6-G(high) antitumor subsets [45]. A more recent study from another group demonstrated that the flagella of *Salmonella* VNP20009 strain can induce activation of Flagellin/TLR5/NF-*κ*B signaling in splenic and intratumoral immune infiltrates and that flagella-deficient *Salmonella* did not elicit substantial antitumor response [46]. Results of their experiments also revealed that T cells from tumors receiving wild-type *Salmonella* enhanced RNA expression of key inflammatory cytokines (i.e., IL-4, IL-5, IL-13, IL-17, IL-21, IL-22 and IFN-*γ*), compared with T cells from tumors treated with flagellum-deficient strains [46].

### 4.2. Innate Immunity

Immune cells of the innate lineage also form part of the inflammatory TME. Since the prevalent immunosuppressive phenotype in tumor-associated macrophages is associated with poor clinical prognosis relating to cancer progression, inducing phenotypic reprogramming may help improve prognosis [47,48]. We already mentioned that *Salmonella*-based treatment plays an essential role in modulating antitumor immune response. Interestingly, this *Salmonella*-mediated immune response not only increases antitumor T cell activity but also entices the participation of macrophages, neutrophils, dendritic, and NK cells [11,40,41].

Our group is interested in deciphering how *Salmonella* reprograms host immunity toward becoming more tumoricidal. In particular, we recently found that *Salmonella* can reprogram tumor-associated macrophages (TAMs) toward an antitumor phenotype [49]. TAMs originate from the myeloid lineage and exist in two forms in the TME, namely, M1 and M2 macrophages, whose subpopulation ratio and prevalence may determine patient outcome [50]. The presence of more M1-like macrophages in a tumor may indicate better clinical outcome. TAMs may be resident macrophages or those that are recruited into the tumor tissue from the bone marrow; these cells normally differentiate from monocytes [51]. The accumulation and phenotype switching of macrophages in the tumor in order to polarize into either M1-like or M2-like phenotype depends on the cytokines and chemokines produced and secreted either by the tumor or stromal cells. Classical activation of TAMs, as stimulated by LPS, IFN-*γ*, TNF-*α,* and HMGB1, generates M1-like phenotypes regarded as antitumor macrophages due to their potential to undermine tumor growth and progression; these macrophages are aggressive in nature and highly phagocytic, and can promote Th1 responses [52,53,54]. On the other hand, the M2-like phenotype is the outcome of alternative activation of TAMs; these cells positively influence the tumor, as they are involved in promoting immunosuppression, angiogenesis, and extracellular matrix (ECM) remodeling [52,53,55]. In our study, we found that *Salmonella* treatment polarizes TAMs toward an M1-like phenotype by increasing tumor secretion of HMGB1 (Figure 1) [49]. The role of HMGB1 in proinflammatory M1-like reprogramming has already been reported, and that this late inflammatory cytokine influences TAMs polarization possibly via multiple pathways such as TLR2, TLR4, and RAGE/NF-κB signaling cascades [56,57]. Polarized M1-like macrophages in the TME, following *Salmonella* treatment, produce significant amounts of proinflammatory cytokine Interleukin-1*β* (IL-1*β*), and that increase in IL-1*β* corresponds to tumor regression [49,58]. An earlier study also revealed that the direct interaction between *Salmonella* and bone marrow-derived macrophages (BMDM) positively influenced production of inflammasome-related proteins such as NLRP3, IPAF and caspase-1 p10, and a high-level secretion of IL-1*β* [59]. Although IL-1*β* appears to be involved in tumor invasion and angiogenesis, its role in the context of cancer treatment has also been reported [60,61,62]. IL-1*β* inhibits tumor growth by promoting tumor cell death; this is accomplished by priming CD8+ T cells or stimulating CD4+ T cells toward Th1 response [58].

## 5. Antitumor Immune Modulation via *Salmonella* Delivery System

Early attempts in *Salmonella*-based cancer treatment for phase 1 clinical trials using the VNP20009 strain failed in inducing significant tumor regression along with documented side effects at high dosage, and resulted in the research being discontinued [63]; nevertheless, the observed increase of circulating inflammation-related cytokines (TNF-*α*, IL-6, IL-1*β*, and IL-12) and tumor colonization noted from biopsies of some patients in the trial [63] prompted scientists and clinicians to improve *Salmonella*-based treatment. Owing to its tumor-targeting, innate oncolytic ability, and immune modulation, *Salmonella* is now considered a viable tool as a vehicle for the delivery of antitumor compounds, RNAi, therapeutic genes, and vaccines via gene-editing or engineering techniques [64,65,66]. The summary of the immunomodulatory effects of *Salmonella* carrying various therapeutic agents are listed in Table 1. Prior accounts utilizing *Salmonella* as a delivery vector for RNA interference targeting the expression of oncogenic products, including STAT3 [67], Bcl-2 [68], P-glycoprotein [69], and HIF-1 [70], has been demonstrated to promote significant tumor regression and increased survival in murine models. This may be explained, in part, by the participation of tumor-secretory cytokines and chemokines that are inhibited by STAT3, which then promotes dendritic cell maturation and T cell activation [21]. This context, however, needs further confirmation. *Salmonella* was also used to deliver shRNA to target the immunosuppressive factor IDO; the IDO blockade turned out to cause significant tumor cell death that is associated with a substantial increase of infiltrating reactive oxygen species (ROS)-producing polymorphonuclear neutrophils (PMN) [71]. Further, several reports suggested that orally delivered cytokine gene therapy using *Salmonella* as the vector—carrying expression vectors encoding IL-12, GM-CSF, and IL-4 or IL-18 genes—significantly increased cytotoxic T cell activity and prolonged mice survival [72,73]. Intravenous administration of *Salmonella* encoding cytokine LIGHT (a member of TNF ligand superfamily) markedly increased CD19+ B cells, CD4+ T cells, and CD8+ T cells in tumors [74]. In another study, treatment with the *Salmonella* encoding IL-2 gene demonstrated a reduced osteosarcoma pulmonary metastasis associated with considerable increase of pulmonary NK cell population [75]. Oral administration of the *Salmonella* encoding IL-2 gene has been subjected to phase 1 clinical study for canine osteosarcoma [76]. Surprisingly, a recent phase 1 clinical trial using *Salmonella* encoding human IL-2 gene was conducted for patients suffering from metastatic gastrointestinal cancer [77]. Findings from this dose-escalation study included an increase in circulating NK cells and NK-T cells compared with the pre-study period, while no toxicity or adverse events were observed during the study period [77]. It was previously mentioned that IFN-*γ* and IFN-inducible chemokines are also mediators that stimulate antitumor immune response [39,40]. Yoon et al. [78] demonstrated that *S. typhimurium* encoding SipB160/IFN-*γ* elicited tumor regression in an NK cell-dependent manner with no observable adverse reactions. An engineered *Salmonella* generated to secrete heterologous flagellin B (FlaB) from *Vibrio vulnificus* was shown to incite immune infiltration via TLR4 signaling, leading to the subsequent reprogramming of macrophages, via TLR5 signaling, toward an M1-like antitumor phenotype [79].

## 6. *Salmonella* as Tool for Anticancer Vaccine

Advances in studying *Salmonella*-based therapy sparked innovation in the development of a therapeutic vaccine for various types of cancer. Primarily, the goal of therapeutic vaccines is to coax lasting antitumor memory apart from eradicating an already established tumor mass [87]. Enhanced infiltration and activation of T cell subsets is essential for an efficacious vaccine. In fact, significant tumor regression was observed in T cell-deficient models following intravenous infusion with tumor-sensitized splenic T cells from immune donors [88,89,90]. Recently, Fan et al. [91] developed a *Salmonella* strain, namely *S. typhimurium* RE88 by H_2_O_2_ inactivation to serve as carrier platform for cancer vaccines. Findings from the study indicated that subcutaneous vaccination with H_2_O_2_-inactivated *Salmonella* enhanced splenic T cell activation and increased CD4+ and CD8+ T cell infiltration into the tumor tissue. In a bacteria-based vaccine platform, cytotoxic T cells are the primary effectors of tumor-specific immune response, while both the CD4+ and CD8+ T cells mediated the memory phase thereafter [92]. *Salmonella* relies on its TTSS to deliver heterologous antigens into the cytosol of affected cells—usually, antigen presenting cells or APCs for optimal immunogenicity [93]. APCs are vital in promoting adaptive immune responses via antigen processing and presentation that is then recognized by T cells [94]. Several DNA vaccines or tumor-associated antigens (TAA) reliant on *Salmonella*-mediated delivery have been studied so far (Table 2), some of which have been approved for early-phase clinical trials. A list of previous and ongoing clinical trials is presented in Table 3. Niethammer et al. [95] reported a double-blind, placebo-controlled, dose-escalation phase I trial of oral DNA vaccine VXM01 (against VEGF receptor 2) carried by *S. typhi* Ty21a for locally advanced and stage IV pancreatic cancer. An early-phase trial revealed that VEGFR2 specific effector T cells, but not Treg responses, were substantially increased among vaccinated patients; this correlates with a reduction of tumor perfusion, an increased serum biomarker for antiangiogenic activity, and increased collagen IV at the 38th day post-vaccination with no recorded dose-limiting toxicities [95,96]. A phase 1 trial extension for up to six monthly boost vaccinations or placebo further demonstrated a pronounced increase in VEGFR2-specific T cell response [97]. Another candidate vaccine generated from a TAA known as Survivin using *Salmonella* as a vector, designated as CVD908ssb-TXSVN, is on its recruiting status for a multiple myeloma phase I clinical trial [98]. More recently, a phase I clinical trial of an oral DNA vaccine using *S. enterica* carrying neuroblastoma-associated antigens in combination with intramuscular injection of DNA-polyethylenimine conjugate is likewise on its recruiting stage [99]. Taken together, these *Salmonella*-based vaccines provide a state-of-the-art therapeutic intervention for cancer treatment and prevention of cancer recurrence and metastasis.

## 7. In Tandem Therapy

The concept of combinatorial treatment has been promoted in the attempt to address documented treatment drawbacks—relating to poor targeting, treatment resistance, and low response among patients—in conventional treatment modalities including chemotherapy, radiation therapy, cytokine therapy, and mAbs. *Salmonella* therapy in combination with radiation therapy such as X-ray provided an additive effect in suppressing tumor growth and prolonging survival in mice bearing B16F10 or Cloudman S91 melanomas [117] and CT26 colon tumors [118]. Furthermore, *γ*-radiation-inducible ROS expression and H_2_AX phosphorylation mediated by *Salmonella* infection substantially increased apoptosis in melanoma [119]. These combinatorial therapies have shown eradication of tumors which may be an outcome of activating radiation-induced tumor cell death. The role of tumor-specific immunity in this context may be investigated further, as a report suggests that the ionizing radiation contributes to immunogenic tumor cell death [120]. Therapeutic combinations with other existing treatment approaches or interventions have also been reported to enhance the effect of *Salmonella*-based treatment in modulating tumor-specific immune responses [120]. For instance, we previously reported that *Salmonella* treatment did enhance tumor chemosensitivity to cisplatin via Cx43 upregulation [121]. The *Salmonella* + cisplatin synergistic effect resulted in a significant tumor regression that is accounted for, in part, by the increased infiltrating neutrophils and CD8+ T cells compared with either *Salmonella* or cisplatin alone [121,122]. Doxorubicin is another chemotherapeutic drug investigated for combination therapy with *S. typhimurium* DSLpNG in the autochthonous model of breast cancer demonstrating significant CD8+ T cell infiltration, but not the Treg population [123]. In another study, *S. typhimurium* LVR01 combined with CHOP chemotherapy (cyclophosphamide, doxorubicin, vincristine, and prednisone/steroid combination) enhanced NK cell-mediated cytotoxic activity along with greater intratumoral CD8+ T cell and neutrophil infiltrations compared with non-combinatorial treatments, revealing the participation of systemic lymphoma-specific humoral immune responses [124]. In contrast, triptolide and *Salmonella* VNP20009 combination relied on enhanced *Salmonella*-mediated direct tumor lysis as triptolide substantially inhibited neutrophil infiltration along with reduced production of proinflammatory cytokines and chemokines (IL-6, IL-1α, IL-1β, IL-12p70, IL-17, IL-13, G-CSF, GM-CSF, MIP-1*α*, MIP-1*β*, KC, and eotaxin) in the TME [125]. Meanwhile, another group combined the *Salmonella*-based treatment with adoptive T cell therapy, which, as expected improved the regression of tumors; the observed outcome was found to be mediated by an increased neutrophil and decreased monocyte population, and secondarily rescued dysfunctional endogenous tumor-specific T cells within the TME [126].

## 8. Conclusions

*Salmonella*-based cancer therapy is indeed a potential and promising approach owing to its innate potential to influence the destruction of cancer cells, and its versatility to act as a vehicle for the delivery of therapeutic genes, drugs, and vaccines. Findings from various research groups, as described herein, suggested that *Salmonella*-based treatments employ the active participation of both the innate and adaptive immune responses. It has been shown that *Salmonella* taps a multilayer of molecular and cellular mechanisms in modulating the immune responses. In particular, *Salmonella* deters the immunosuppressive nature of the TME by altering tumor-intrinsic signaling cascades and the overall gene expression pattern. This is coupled with enhanced infiltration and activation of tumor-specific immune cells, including macrophages, neutrophils, NK cells, CD4+, and CD8+ T cells, and/or the reactivation of dysfunctional effector cells. It is also noteworthy to mention that *Salmonella* can serve as an efficient platform for gene therapy, RNAi, cytokine therapy and vaccine delivery, to address not only an already established tumor but also to prevent disease recurrence and metastasis. The growing plethora of evidence has shown that this innovative strategy for the treatment of cancer holds a potential for routine clinical application that overcomes the limitations of conventional therapies.

## Figures and Tables

**Table 1:** *Salmonella* as a delivery vehicle for cancer immunotherapy.

Strains	Cargo	Immune Modulation Effects	Cell Lines	Mouse Models	Ref.
*S. typhimurium*	LIGHT	Increased splenic CD11c + CD205+ DCs; CXCR3-mediated increase of tumor infiltration with T cells	CT-26 colon carcinoma cells; Lewis lung carcinoma cells	BALB/c; C57BL/6 mice	[74]
*S. typhimurium*	Fas ligand (FasL)	Neutrophil-dependent antitumor activity	CT-26 colon carcinoma cells; D2F2 breast cancer; B16F10 melanoma	BALB/c; C57BL/6 mice	[80]
*S. typhimurium*	IL-18	Increased cytokine secretion by tumors; enhanced infiltration and accumulation of T cells, NK cells, and granulocytes	CT-26 colon carcinoma cells; D2F2 breast cancer; Lewis lung carcinoma cells	BALB/c; C57BL/6 mice	[81]
*S. typhimurium* SalpIL2	IL-2	Increased NK cell population in both spleen and metastatic tumor mass	Osteosarcoma cells	BALB/c mice	[75]
*S. typhimurium* (pur-/msb-)	CCL21	Elevated CXCL9, CXCL10, and IFN-*γ* in the TME; abundant mononuclear and polynuclear cell tumor infiltrates	CT-26 colon carcinoma and B16F10 melanoma cells	BALB/c; C57BL/6 mice	[82]
*S. typhimurium*	IDO shRNA	Recruits ROS-producing PMNs	B16F10 melanoma cells	C57BL/6, IDO-KO; Rag1-KO mice	[71]
*S. typhimurium* VNP20009	PNP	Enhances CD8(+) T-cell infiltration	B16F10 melanoma cells	C57BL/6J mice	[83]
*S. typhimurium* BRD509	HPV16 E7	Increased serum IFN-*γ* and TNF-*α*; enhanced CTL activity	TC-1 cervical cancer cells	C57BL/6J mice	[84]
*S. typhimurium* SHJ2037	FlaB	TLR5-dependent infiltration, and activation of immune cells thereafter	MC38; B16F10 cells; TLR5–negative colon cancer cells	TLR4−/−, TLR5−/−, and MyD88−/− KO mice (C57BL/6 genetic background)	[79]
*S. typhimurium* BRD509	IFN-γ	Enhanced IFN-*γ*-mediated NK cell activity	B16F10 and A375SM human melanoma cells	C57BL/6 mice; RAG0.γc0 lacking NK cells (C57BL/6 background)	[78]
*S. typhimurium* BRD509	IL-2	NK and cytotoxic T cell-mediated tumor apoptosis	B16F10 melanoma cells	C57BL/6J mice	[85,86]

**Table 2:** *Salmonella*-based cancer vaccine platforms.

Species	Route	Cargo	Murine Cancer Type	Ref.
*S. typhimurium-lux*	Oral	Mouse α-fetoprotein gene	Colon carcinoma; Hepatoma	[100]
*S. typhimurium* (ΔphoP ΔphoQ)	Oral; Intratumoral	NY-ESO-1 tumor antigen	Sarcoma	[93]
*S. typhimurium* (Dam−; AroA−)	Oral	Legumain	Breast cancer	[101]
*S. typhimurium* SL3261	Oral	VEGFR-2/FLK1	Lung carcinoma	[102]
*S. typhimurium* MvP728 (purD/htrA)	Oral	Survivin; MYCN oncoproteins	Colon carcinoma; Glioblastoma; B cell lymphoma	[103,104,105]
*S. typhimurium* SL7207	Oral	Mouse prostate stem cell antigen	TRAMPC1 prostate carcinoma	[106]
*S. typhimurium* SL7207	Oral	Survivin	Neuroblastoma	[107]
*Salmonella* SL3261	Oral	4-1BBL	Colorectal cancer	[108]
*S. typhimurium* A1-R	Intravenous	Tumor-specific antigen ovalbumin (OVA)	Melanoma	[109]
*S. typhimurium* SL7207	Intranasal	AIDA-1 autotransporter and DNA vaccine elements	Melanoma	[110]

**Table 3:** Previous and ongoing clinical trials for *Salmonella*-based cancer therapy.

Strains	Cargo	Route	Cancer Type	Phase and Status	No. of Enrolled Patients	NCT Number	Ref.
VNP20009	n/a	Intratumoral	Neoplasm, metastatic	Phase I, completed	45	NCT00004988	[111]
VNP20009	n/a	Intratumoral	Metastatic melanoma and renal cancer	Phase I, completed	45	NCT00006254	[112]
VNP20009	n/a	Intratumoral	Unspecified adult solid tumors (advanced/metastatic)	Phase I, completed	40	NCT00004216	[113]
*S. typhimurium* SalpIL2	human IL-2	Oral	Solid tumors (unresectable hepatic spread)	Phase I, completed	22	NCT01099631	[114]
*S. typhimurium* Ty21a (VXM01 vaccine)	VEGFR2	Oral	Advanced pancreatic cancer	Phase I, completed	72	NCT01486329	[115]
*Salmonella* CVD908ssb (TXSVN vaccine)	Survivin	Oral	Multiple myeloma	Phase I, recruiting	24 (est.)	NCT03762291	[98]
*S. typhimurium* SS2017	Tumor-associated antigens	Oral	Neuroblastoma	Early phase I, recruiting	12 (est.)	NCT04049864	[99]
*S. typhimurium* Saltikva	human IL-2	Oral	Metastatic pancreatic cancer	Phase II, recruiting	60 (est.)	NCT045892	[116]

Note: n/a, not applicable; est., estimated.

## Data Availability

Not applicable.

## Abbreviations

4-1BBL	TNF ligand superfamily member 9
AIDA-1	Adhesin involved in diffuse adherence
Akt	Protein kinase B
APC	Antigen-presenting cells
ARG1	Arginase 1
BMCT	Bacteria-mediated cancer therapy
CCL	Chemokine (C-C motif) ligand
CD	Cluster of differentiation
CTL	Cytotoxic T cells
Cx43	Connexin 43
CXCL	Chemokine (C-X-C motif) ligand
CXCR	Chemokine receptor
DCs	Dendritic cells
ECM	Extracellular matrix
FlaB	Flagellin B
FLK1	Fetal Liver Kinase-1
HMGB1	High mobility group box 1 protein
HPV16 E7	Human papillomavirus type 16 protein E7
IDO	Indoleamine-2,3-dioxygenase
IFN-*γ*	Interferon-gamma
IL	Interleukins
iNOS	Inducible nitric oxide synthase
LIGHT	Lymphotoxin-like inducible protein that competes with glycoprotein D for herpes virus entry on T cells
KO	Knockout
LPS	Lipopolysaccharide
mTOR	Mammalian target of rapamycin
MYCN	MYCN protooncogene
MyD88	Myeloid differentiation primary response 88
NK	Natural killer
NY-ESO-1	New York esophageal squamous cell carcinoma 1
PD-1	Programmed Death-1
PD-L1	Programmed Death-Ligand 1
PNP	Purine nucleoside phosphorylase
PMNs	Polymorphonuclear leukocytes
shRNA	Short hairpin ribonucleic acid
TAA	Tumor-associated antigens
TAMs	Tumor-associated macrophages
TLR	Toll-like receptor
TNF-*α*	Tumor necrosis factor-alpha
TME	Tumor microenvironment
Treg	Regulatory T cells
TTSS	Type three secretion system
VEGF	Vascular endothelial growth factor
